# Vanillin Protects Dopaminergic Neurons against Inflammation-Mediated Cell Death by Inhibiting ERK1/2, P38 and the NF-κB Signaling Pathway

**DOI:** 10.3390/ijms18020389

**Published:** 2017-02-12

**Authors:** Xuan Yan, Dian-Feng Liu, Xiang-Yang Zhang, Dong Liu, Shi-Yao Xu, Guang-Xin Chen, Bing-Xu Huang, Wen-Zhi Ren, Wei Wang, Shou-Peng Fu, Ju-Xiong Liu

**Affiliations:** 1 College of Animal Science and Veterinary Medicine, Jilin University, Changchun 130062, China; yanxuan1992@outlook.com (X.Y.); ccdf@163.com (D.-F.L.); DE81445@163.com (X.-Y.Z.); xushiyao1989@163.com (S.-Y.X.); gxchen51143@163.com (G.-X.C.); huangbingxu123@163.com (B.-X.H.); rwz1964@163.com (W.-Z.R.); wang_wei99@jluhp.edu.cn (W.W.); 2 Animal Husbandry and Veterinary Medicine, Cangzhou Technic College, Cangzhou 061001, China; dodo_cz@163.com

**Keywords:** vanillin, inflammation, microglia, Parkinson’s disease, MAPK, NF-κB

## Abstract

Neuroinflammation plays a very important role in the pathogenesis of Parkinson’s disease (PD). After activation, microglia produce pro-inflammatory mediators that damage surrounding neurons. Consequently, the inhibition of microglial activation might represent a new therapeutic approach of PD. Vanillin has been shown to protect dopaminergic neurons, but the mechanism is still unclear. Herein, we further study the underlying mechanisms in lipopolysaccharide (LPS)-induced PD models. In vivo, we firstly established rat models of PD by unilateral injection of LPS into substantia nigra (SN), and then examined the role of vanillin in motor dysfunction, microglial activation and degeneration of dopaminergic neurons. In vitro, murine microglial BV-2 cells were treated with vanillin prior to the incubation of LPS, and then the inflammatory responses and the related signaling pathways were analyzed. The in vivo results showed that vanillin markedly improved the motor dysfunction, suppressed degeneration of dopaminergic neurons and inhibited microglial over-activation induced by LPS intranigral injection. The in vitro studies demonstrated that vanillin reduces LPS-induced expression of inducible nitric oxide (iNOS), cyclooxygenase-2 (COX-2), IL-1β, and IL-6 through regulating ERK1/2, p38 and NF-κB signaling. Collectively, these data indicated that vanillin has a role in protecting dopaminergic neurons via inhibiting inflammatory activation.

## 1. Introduction

Parkinson’s disease (PD) is the second most common neurodegenerative disorder that affects millions of people in the world [1,2,3]. It is characterized by progressive degeneration of dopaminergic neurons in the substantia nigra pars compacta (SNpc), resulting in a substantial loss of dopamine afferents to the striatum and subsequent motor impairment. Although the precise pathophysiology that precipitates the development of PD is unknown, accumulating evidence suggested a potential role of neuroinflammation in the pathogenesis of PD [4,5,6,7]. Although microglia represent only 5%–20% of the Central Nervous System (CNS) cells, they act as the main effector cells on the procession of neuroinflammation [8,9]. Microglia are the main participants of neuroinflammation. Over-active microglia can produce a large amount of inflammatory mediators, which are involved in the progression of neuronal degeneration in PD [10,11]. Therefore, the inhibition of microglia activation may be a potential therapeutic strategy for PD.

Vanillin (4-hydroxy-3-methoxybenzaldehyde) is the major component of natural vanilla, which is one of the most widely used flavor components in food and personal products [12]. Besides its flavor qualities, vanillin exhibits the anti-microbial, anti-mutagenic, anti-angiogenetic effects [13,14]. Dhanalakshmi et al. have found that vanillin plays an important role in the rotenone-induced rat model of PD and protects against rotenone-induced neurotoxicity in SH-SY5Y cells, suggesting that vanillin is a potential neuroprotective agent in PD models, but the exact mechanism remains unclear [15,16]. Lots of studies have shown that vanillin has anti-inflammatory effects in peripheral tissues, such as liver, colon and macrophages [17,18,19]. However, no reports showed whether vanillin has an anti-inflammatory effect on the central nervous system. Herein, we hypothesize that vanillin might have a potential role in inhibiting microglial over-activation and then alleviates further PD development. In this study, we aim to investigate the neuroprotective and anti-inflammatory properties of vanillin in lipopolysaccharide (LPS)-induced in vivo and in vitro PD models so as to reveal the mechanism of vanillin in inhibiting inflammatory response.

## 2. Results

### 2.1. Vanillin Treatment Improves Motor Dysfunction Induced by LPS Intranigral Injection

**Figure 1:**
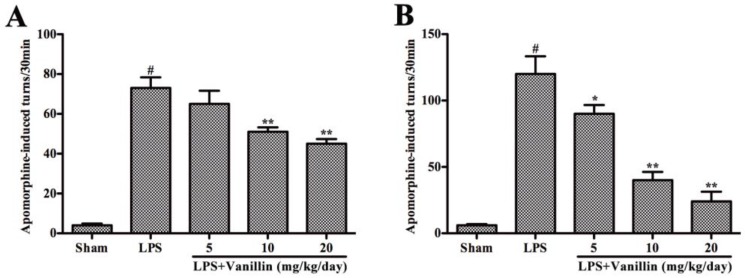
Vanillin administration improves the motor dysfunction by lipopolysaccharide (LPS) intranigral injection. Rats were treated with the vehicle or vanillin (5, 10, or 20 mg/kg/day) for three days prior to LPS intranigral injection and this lasted for 24 days. The number of turns after two (**A**); and four weeks (**B**) was induced by apomorphine in Parkinson’s disease (PD) model rats. # *p* < 0.01 vs. the sham-operated control rats; and * *p* < 0.05 and ** *p* < 0.01 vs. the vehicle treated LPS-injected rats.

LPS, which is injected into unilateral SN, leading to microglial activation and the damage of dopaminergic neurons. Unilateral injury of dopaminergic neurons resulted in the compensatory hyper function of dopamine receptors. Due to the different receptor activity on both sides, the receptor agonist leads to the rotation behavior towards the injection side. The therapeutic effects of drugs on lesion degree in PD animal models are characterized by a rotational behavior assay. In order to examine the effect of vanillin administration on motor dysfunction, after LPS injection, the rats were subjected to rotational behavior assay at two and four weeks. Notably, vanillin dose-dependently attenuated apomorphine-induced rotation (Figure 1). Moreover, the role of vanillin in the fourth week is more effective than the second week (Figure 1).

### 2.2. Vanillin Adminastration Increases the Survival Rate of Dopaminergic Neurons in the SN

**Figure 2:**
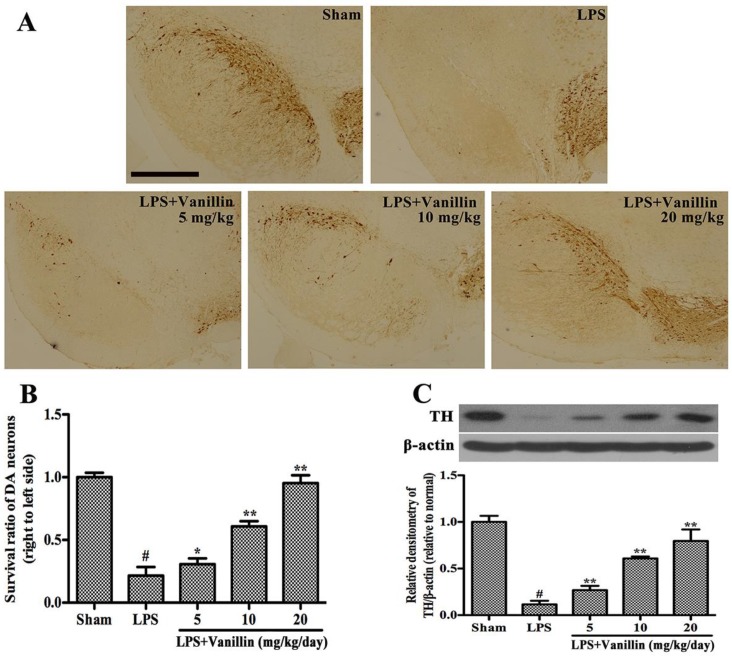
Vanillin administration increases the survival rate of dopaminergic neurons in the substantia nigra (SN). The PD model rats were anaesthetized and decapitated to obtain the SN after 24 days of vanillin administration. (**A**) Immunohistochemical results of tyrosine hydroxylase (TH)-positive cells, scale bar represents 1.0 mm; (**B**) The survival ratio of the dopaminergic neurons; and (**C**) TH expression. # *p* < 0.01 vs. the sham-operated control rats; and. * *p* < 0.05 and ** *p* < 0.01 vs. the vehicle treated LPS-injected rats.

In order to investigate whether vanillin protects dopaminergic neurons, the number of tyrosine hydroxylase (TH)-positive neurons and the expression of TH were carried out by immunohistological and Western blot analysis respectively. As the result has showed, compared with sham-operated rats, LPS-injected rats have significantly reduced TH-positive neurons in the SN section (*p* < 0.01). In contrast, vanillin (5, 10, or 20 mg/kg/day) administration markedly increased the number of TH-positive neurons in a dose-dependent manner (Figure 2A,B). The results of Western blot showed that there is significant reduction in TH expression in the SN after LPS injection. Vanillin treatment significantly increased the expression of TH in a dose-dependent manner (Figure 2C). Thus, vanillin can protect dopaminergic neurons in the SN of LPS-induced PD model rats.

### 2.3. Vanillin Inhibits LPS-Induced Activation of Microglia in the SN 

**Figure 3:**
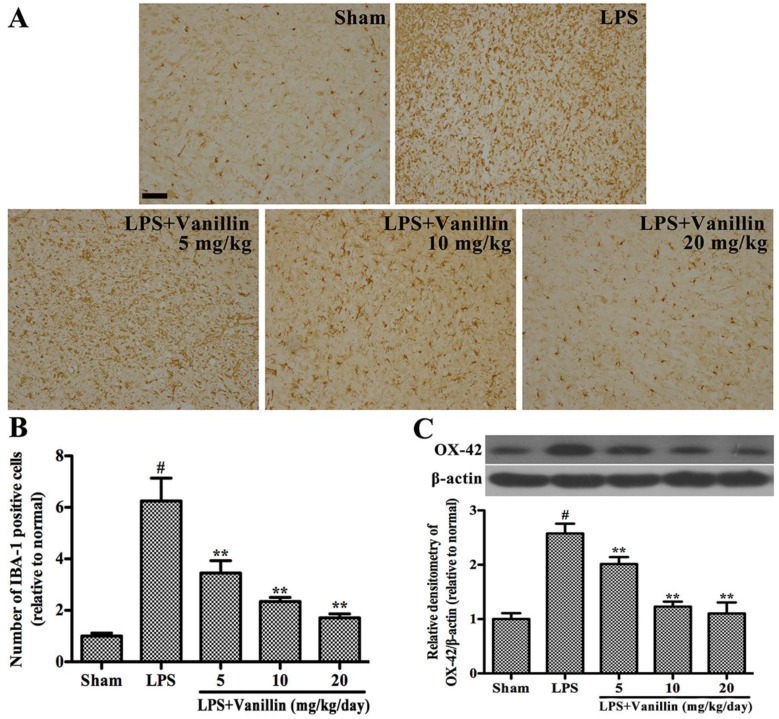
Vanillin inhibits LPS-induced activation of microglia in the SN. The PD model rats were anaesthetized and decapitated to obtain the SN after 24 days of vanillin administration. (**A**) immunohistochemical results of ionized calcium-binding adaptor molecule-1 (IBA-1)-positive cells, scale bar represents 100 μm; (**B**) the number of IBA-1-positive cells; (**C**) O-X42 expression. # *p* < 0.01 vs. the sham-operated control rats; and. ** *p* < 0.01 vs. the vehicle treated LPS-injected rats.

In order to clarify whether the anti-inflammation is associated with protecting dopaminergic neurons, we further examined the effect of vanillin on LPS-induced activation of microglia in the SN. Microglia Activation was characterized by ionized calcium-binding adaptor molecule-1 (IBA-1) [20]. IBA-1-positive cells ramified resting microglia with two or three fine processes in the SN of sham-operated rats (Figure 3A). After LPS intranigral injection and treatment with the vehicle (normal saline), the number of microglia markedly increased (Figure 3A) and the rest ramified microglia were transformed into an amoeboid morphology (Figure 3B). After LPS injection plus treatment with vanillin, microglial activation was dramatically inhibited in a concentration dependent manner (Figure 3A,B). In order to further prove whether vanillin treatment inhibits microglial activation, we examined the expression of OX-42 in the SN of LPS-induced PD model rats by Western blot. The results showed that LPS intranigral injection markedly increased the expression of OX-42, and vanillin administration significantly decreased its expression in a concentration dependent manner (Figure 3C).

### 2.4. Vanillin Inhibits Inflammatory Responses Induced by LPS in BV-2 Cells

**Figure 4:**
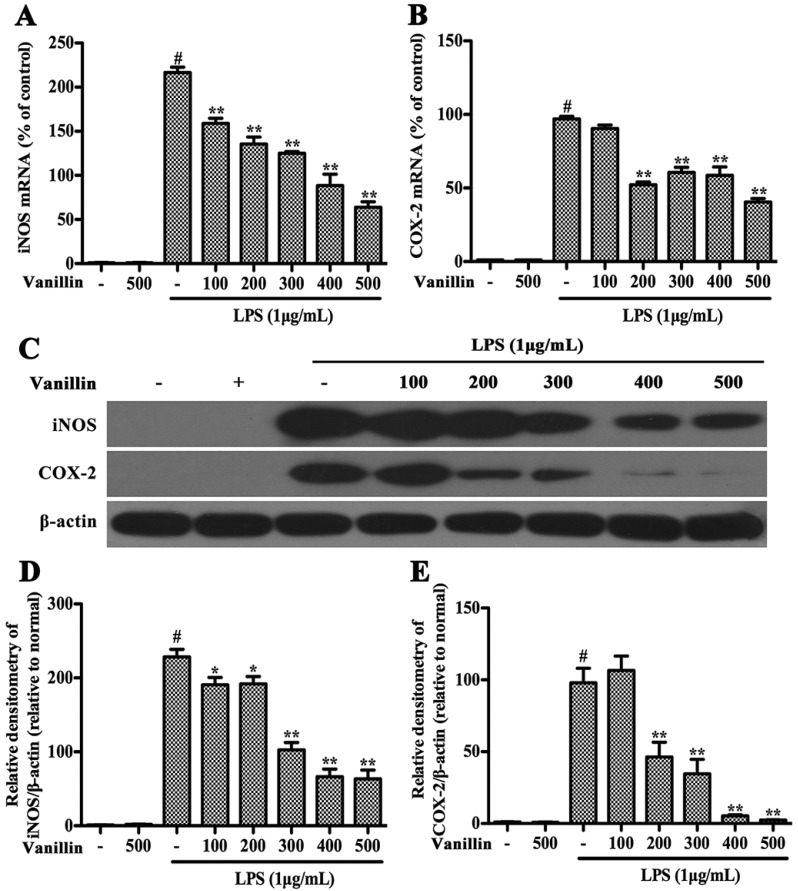
Vanillin attenuates the production of inducible nitric oxide (iNOS) and cyclooxygenase-2 (COX-2) induced by LPS in BV-2 cells. Cells were pretreated with vanillin (100, 200, 300, 400 and 500 nM) for 1 h and were then incubated with LPS (1 µg/mL) for 4 h. The mRNA levels of iNOS (**A**); and COX-2 (**B**) in BV-2 cells were examined by quantitative real-time PCR. The protein levels of iNOS (**C**,**D**) and COX-2 (**C**, **E**) were determined by Western blot. The data were normalized to β-actin. # *p* < 0.01 vs. no treated group. * *p* < 0.05 and ** *p* < 0.01 vs. the vanillin-untreated LPS-stimulated group.

**Figure 5:**
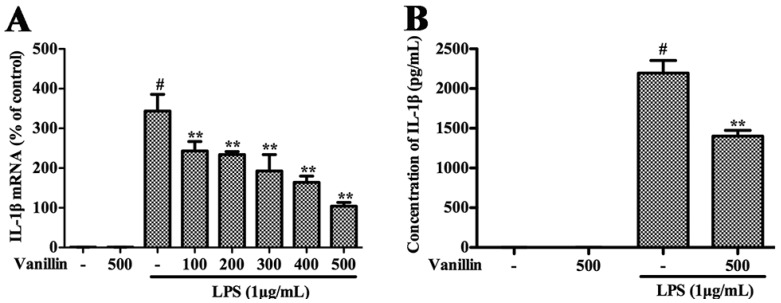

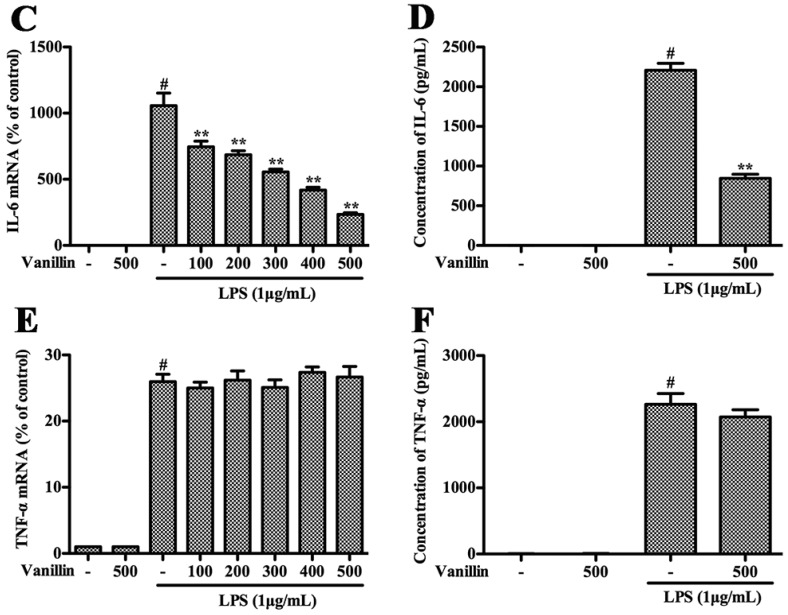
Effects of vanillin on LPS-induced proteins production and mRNA expression of pro-inflammatory cytokines in BV-2 cells were examined. The mRNA levels of IL-1β (**A**), IL-6 (**C**) and TNF-α (**E**) in BV-2 cells were examined by quantitative real-time PCR. Cells were pretreated with vanillin (100, 200, 300, 400 and 500 nM) for 1 h, and then incubated with LPS (1 µg/mL) for 4 h; the expression level of IL-1β (**B**), IL-6 (**D**) and TNF-α (**F**) was determined via ELISA. Cells were pretreated with vanillin (500 nM) for 1 h, and then incubated with LPS (1 µg/mL) for 12 h. # *p* < 0.01 vs. no treated group. ** *p* < 0.01 vs. the vanillin-untreated LPS-stimulated group.

Henn et al. have proved that BV-2 cells are the most commonly used alternative to primary microglia [21]. Therefore, we examined vanillin’s anti-inflammatory function in LPS-induced BV-2 cells. In BV-2 cells, LPS could increase the production of pro-inflammatory cytokines (TNF-α, IL-1β, and IL-6) and pro-inflammatory enzymes (iNOS and COX-2), which play very important roles in the process of inflammation [22]. In order to examine the effect of vanillin administration on the inflammatory responses induced by LPS in BV-2 cells, BV-2 cells were pretreated with vanillin (100, 200, 300, 400 and 500 nM) for 1 h and then incubated with LPS (1 µg/mL) for different time periods, and the mRNA and protein levels of these pro-inflammatory mediators were detected by quantitative real-time PCR and Western blot, respectively. The results showed that vanillin markedly inhibited LPS-induced increase in the mRNA and protein levels of iNOS (Figure 4A,C,D), COX-2 (Figure 4B,C,E), IL-1β (Figure 5A,B) and IL-6 (Figure 5C,D) in a concentration dependent manner, while no effect was observed in TNF-α (Figure 5E,F). To examine the cytotoxicity of vanillin, BV-2 cells were stimulated with different doses of vanillin for 24 h, and then the cell viability was detected by MTT (3-(4,5-dimethylthiazol-2-yl)-2,5-diphenyltetrazolium bromide) assay. MTT assay results excluded non-specific cytotoxicity of vanillin (data not shown), demonstrating that vanillin in noncytotoxic levels suppressed LPS-induced inflammatory responses in BV-2 cells via inhibiting the production of iNOS, COX-2, IL-1β and IL-6.

### 2.5. Vanillin Inhibits Activation of ERK1/2, p38 and NF-κB Induced by LPS in BV-2 Cells 

**Figure 6:**
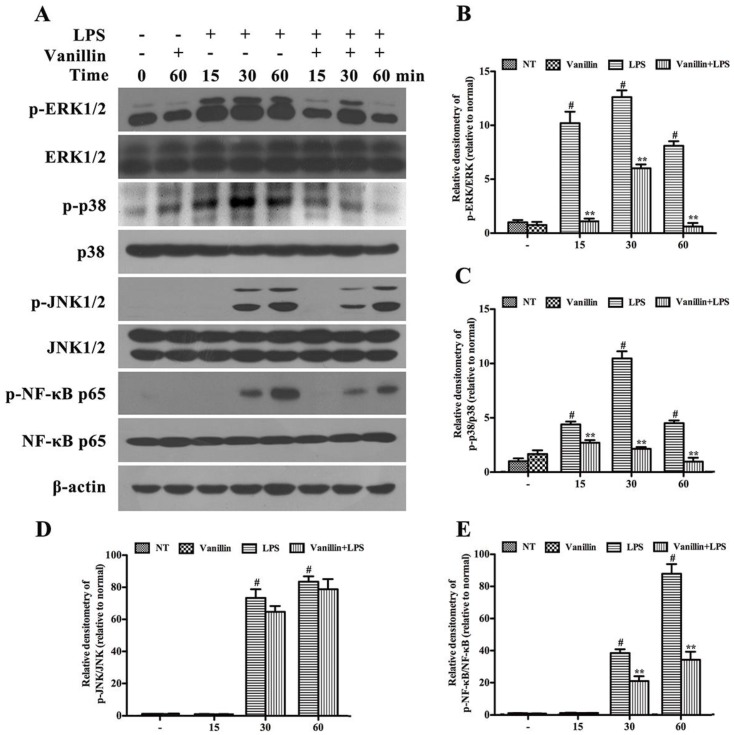
Vanillin regulates MAPKs and NF-κB activation in LPS-stimulated BV-2 cells. (**A**) the expression of MAPKs and NF-κB p65 was examined via Western blot. The phosphorylation ratio of ERK1/2 (**B**); p38 (**C**); JNK1/2 (**D**); and NF-κB p65 (**E**) was quantified. Each value was then expressed relative to the control (LPS no treatment), which was set as 1.00. # *p* < 0.01 vs. no control. ** *p* < 0.01 vs. treated with LPS alone within the same time point.

MAPKs and NF-κB play a pivotal role in modulating the expression of pro-inflammatory mediators in LPS-induced microglia [23,24,25]. To further elucidate how vanillin modulated microglial activation, the phosphorylation of MAPKs and NF-κB p65 was detected in LPS-treated BV-2 cells post vanillin treatment. Cells were treated with vanillin for 1 h followed by LPS (1 µg/mL) treatment for 15, 30 and 60 min. The results of Western blot showed that the phosphorylation of ERK1/2, p38, JNK1/2 and NF-κB p65 was markedly increased after LPS (1 µg/mL) stimulation in a time-dependent manner (Figure 6A). LPS-induced phosphorylation of ERK1/2 (Figure 6A,B), p38 (Figure 6A,C) and NF-κB p65 (Figure 6A,E) was significantly reduced after vanillin treatment in BV-2 cells, while no obvious changes were observed upon phosphorylation of JNK1/2 (Figure 6A,D).

## 3. Discussion

The finding of our present study showed that vanillin protects dopaminergic neurons via reducing microglial inflammatory response in LPS-induced PD rat models. Further study found that vanillin significantly inhibits subsequent production of pro-inflammatory mediators IL-1β, IL-6, iNOS and COX-2, and eventually inhibited the over-activation of microglia. The mechanistic studies found that vanillin suppresses microglial activation via inhibiting phosphorylation of ERK1/2, p38 and NF-κB p65.

Vanillin is the main constituent of natural vanilla, which plays an important role in treating neurodegenerative disorders, such as Huntington's disease [26]. Many studies have shown that vanillin protects against toxin-induced death of dopaminergic neurons. In vivo, vanillin attenuated the motor and non-motor deficits, neurochemical deficits, oxidative stress and apoptosis caused by rotenone administration in the brain of the rats [16]. Chinnasamy et al. found that vanillin attenuated rotenone-induced neurotoxicity in SH-SY5Y cells [15]. These pieces of data demonstrated that vanillin has an important role in treating PD models, but the exact underlying mechanisms remain unclear.

A large number of studies have shown that neuroinflammation plays a very important role in the pathogenesis of PD [20,22,24]. Necropsy studies found that there are many activated microglia in the SN of PD patients [27]. A large number of activated microglia also found in the SN of PD animal models, such as MPTP- and 6-OHDA-induced PD animal models [28,29]. Experimentally, LPS has been proven to cause damage to the SN, resulting in PD [30,31]. LPS-induced PD rat model is widely used to study the effect of neuroinflammation on the dopaminergic system. Vanillin has anti-inflammatory effects in peripheral tissues. To illustrate the neuroprotective action of vanillin involved in anti-inflammatory function, we examined vanillin impact on the motor dysfunction of LPS-induced PD model rats. LPS, which inject into unilateral SN leading to microglial activation and damage of dopaminergic neurons. Unilateral injury of dopaminergic neuron resulted in the compensatory hyper function of dopamine receptors. Due to the different receptor activity on both sides, the receptor agonist leads to the rotation behavior. Therefore, the damage degree of dopaminergic system was widely assessed by apomorphine-induced rotation behavior. We found that vanillin significantly reduced apomorphine-induced rotation in a dose-dependent manner. The result of TH immunohistological analysis demonstrated that vanillin administration markedly inhibited LPS-induced death of dopaminergic neuron. Further research showed that vanillin inhibited LPS-induced activation of microglia. These results indicated that the neuroprotection effect of vanillin on LPS-induced PD rat model is closely associated with inhibiting activation of microglia.

BV-2 cells deriving from raf/myc-immortalised murine neonatal microglia are the alternative model system for primary microglia cultures or for animal experiments examining brain inflammation [32]. They have been widely used for studying neuroinflammation [23,33]. LPS-induced inflammatory response of BV-2 cells was used to study anti-inflammatory effects of vanillin. We found that LPS significantly increased the expression of pro-inflammatory mediators, and vanillin markedly reduced that. These results further confirmed that the neuroprotection effect of vanillin is involved in its anti-inflammatory function.

MAPKs and NF-κB are important mediators in inflammation. MAPKs take part in regulating the pro-inflammatory cytokines production [34]. NF-κB is also related to regulating the transcription of numerous pro-inflammatory mediators, including iNOS, COX-2, TNF-α, IL-1β, and IL-6 [22]. To confirm how vanillin was involved in the anti-inflammatory reaction, we further investigated the impact of vanillin on p38, ERK1/2, JNK1/2 and NF-κB p65 phosphorylation. The results obtained in this study showed that vanillin treatment markedly inhibits LPS-induced p38, ERK1/2 and NF-κB p65 phosphorylation, but has no effect on phosphorylation of JNK1/2, demonstrating that vanillin suppress the inflammatory response via inhibiting ERK1/2, p38 and NF-κB p65 phosphorylation.

## 4. Materials and Methods

### 4.1. Animals and Surgery

Wistar rats, male (250 to 290 g) were obtained from the Center of Experimental Animals of the Baiqiuen Medical College of Jilin University (Changchun, China). Studies were performed in accordance with the guidelines established by the Jilin University Institutional Animal Care and Use Committee (approved on 27 February 2015, Protocol No. 2015047). The procedure of surgery is as described previously [20]. Briefly, rats were anesthetized with sodium pentobarbital (45 mg/kg, intraperitoneal administration) and positioned in a stereotaxic apparatus, and subsequently received the LPS-injection with total volume of 2 µL (5 µg/µL) or cerebrospinal fluid (CSF) into the right SNpc at a rate of 0.2 µL/min.

### 4.2. Administration of Vanillin

Rats were randomly divided into five groups: the sham-operated group and the LPS-injected groups including vehicle treatment group, 5, 10, or 20 mg/kg/day vanillin (Sigma-Aldrich, St. Louis, MO, USA) treatment group. Vanillin was dissolved in normal saline and intraperitoneally administrated at a dose of 5, 10, or 20 mg/kg once a day since 3 days prior to LPS injection and for 24 days totally.

### 4.3. Apomorphine-Induced Rotational Behavior

Followed LPS (Sigma-Aldrich) injection, rats were intraperitoneally injected with apomorphine to assess lesion severity and rotational behavior assay was performed on the second and the fourth weeks as previously described [20]. Briefly, rats were placed in a circular test arena and allowed to adapt for a short period to the testing environment, injected with dopamine receptor agonist apomorphine. The number of turns was noted 5 min after apomorphine administration and recorded a total 30 min testing period.

### 4.4. Cell Treatments

The BV-2 cells were grown in DMEM (Gibco Life Technologies, Inc., Grand Island, NY, USA) containing 10% FBS (Hyclone, Logan, UT, USA), 50 µg/mL streptomycin and 50 U/mL penicillin. The cells were passaged at 80% confluence after trypsinisation (0.05%, *w*/*v*) and seeded on 6 cm cell culture plate or a 24-well plate. BV-2 cells were cultured in serum-free DMEM for 4 h to reduce mitogenic effects and were then treated with various concentrations of Vanillin for 1 h prior to LPS (1 µg/mL) treatment.

### 4.5. RNA Extraction and Quantitative Real-Time PCR

Total RNA was extracted from BV-2 cells using Trizol (Invitrogen, Carlsbad, CA, USA), and reverse transcription-PCR to cDNA using a commercial RT-PCR Kit (Takara Shuzo Co., Ltd., Kyoto, Japan). The mRNA levels of various genes were evaluated by quantitative real-time PCR using a SYBR Green PCR Master Mix (Roche, South San Francisco, CA, USA), and each sample was analysed in triplicate. The levels of cDNA were calculated relative to *β-actin* using the comparative cycle threshold method. The primer sequences were shown in Table 1.

### 4.6. ELISA

BV-2 cells were seeded in a 24-well plate and treated with Vanillin at various concentrations for 1 h, and then followed by LPS (1 µg/mL) stimulation for another 12 h. Subsequently, the culture medium was collected and centrifuged at 13,000 rpm for 3 min. The production of TNF-α, IL-1β, and IL-6 in the culture medium was analyzed followed the BioLegend (San Diego, CA, USA) manufacturer’s instructions.

### 4.7. Immunohistological Analysis

The midbrains were fixed and processed for immunostaining as described previously [20]. Immunohistological analysis was performed using anti-TH (1:1000; Abcam, Cambridge, CA, USA) and IBA-1 (1:200, Proteintech, Chicago, IL, USA). Three researchers counted the number of TH- and IBA-1 positive cells in SN, and the average of these scores were reported.

### 4.8. Western Blot Analysis

After the behavioral test, the SNs of the rats were rapidly dissected out and homogenized in lysis buffer (Beyotime Inst. Biotech, Beijing, China). The microglial cells were collected and lysed with a lysis buffer. Supernatants were collected and the protein concentrations were measured using a bicinchoninic acid protein assay kit (Beyotime Inst. Biotech). A total of 30 µg of protein was subjected to 10% sodium dodecylsulfate polyacrylamide gel electrophoresis (SDS-PAGE) and transferred onto immunoblot polyvinylidene difluoride membranes (Millipore, Billerica, MA, USA). After blocking with 5% nonfat milk, the blots were incubated overnight at 4 °C with primary antibodies against iNOS (1:2000), COX-2 (1:1000), OX-42 (1:1000), TH (1:1000) (Abcam), phosphor-ERK1/2 (1:2000), ERK1/2 (1:2000), phosphor-p38 (1:2000), p38 (1:1000), phosphor-JNK (1:1000), JNK (1:2000), phosphor-NF-κB p65 (1:1000), NF-κB p65 (1:1000) (Cell Signaling Technology, Danvers, MA, USA), and β-actin (1:2000) (Santa Cruz Biotechnology Inc., Santa Cruz, CA, USA). After this, the blots were incubated with a horseradish peroxidase-labeled secondary goat anti-rabbit (1:2000; Santa Cruz, CA, USA) or rabbit anti-goat antibody (1:2000; Santa Cruz, CA, USA) for 1 h at room temperature. 

### 4.9. Statistical Analyses

The data were analyzed using SPSS 12.0 statistical software package (SPSS Inc., Chicago, IL, USA) and presented as the mean ± SD. The groups were assessed with ANOVA followed by the least significant difference test. A *p* < 0.05 was considered statistically significant.

## 5. Conclusions

Collectively, this study revealed that vanillin treatment improved LPS-induced motor dysfunction and protected dopaminergic neurons via preventing microglial activation. This study revealed a new mechanism by which vanillin exerts its neuroprotective function on PD. Based on these data, we suggested that vanillin might be a strong candidate for the treatment of PD.

## Figures and Tables

**Table 1:** The primer sequences of *β-actin*, inducible nitric oxide (*iNOS*), cyclooxygenase-2 (*COX-2*), *TNF-α*, *IL-1β*, and *IL-6*.

Gene	Sequences	Length (bp)
*β-actin*	(F) 5′-GTCAGGTCATCACTATCGGCAAT-3′ (R) 5′-AGAGGTCTTTACGGATGTCAACGT-3′	147
*iNOS*	(F) 5′-CACCCAGAAGAGTTACAGC-3′ (R) 5′-GGAGGGAAGGGAGAATAG-3′	186
*COX-2*	(F) 5′-AGAGTCAGTTAGTGGGTAGT-3′ (R) 5′-CTTGTAGTAGGCTTAAACATAG-3′	170
*TNF-α*	(F) 5′-CCACGCTCTTCTGTCTACTG-3′ (R) 5′-GCTACGGGCTTGTCACTC-3′	145
*IL-1β*	(F) 5′-TGTGATGTTCCCATTAGAC-3′ (R) 5′-AATACCACTTGTTGGCTTA-3′	131
*IL-6*	(F) 5′-AGCCACTGCCTTCCCTAC-3′ (R) 5′-TTGCCATTGCACAACTCTT-3′	156

## Abbreviations

PD	Parkinson’s disease
LPS	lipopolysaccharide
SN	substantia nigra
SNpc	substantia nigra pars compacta
TH	tyrosine hydroxylase
IBA-1	ionized calcium-binding adaptor molecule-1
